# A Thermodynamic Cycle to Predict the Competitive Inhibition Outcomes of an Evolving Enzyme

**DOI:** 10.1021/acs.jctc.5c00193

**Published:** 2025-04-23

**Authors:** Ebru Cetin, Haleh Abdizadeh, Ali Rana Atilgan, Canan Atilgan

**Affiliations:** Faculty of Engineering and Natural Sciences, Sabanci University, 34956 Istanbul, Türkiye

## Abstract

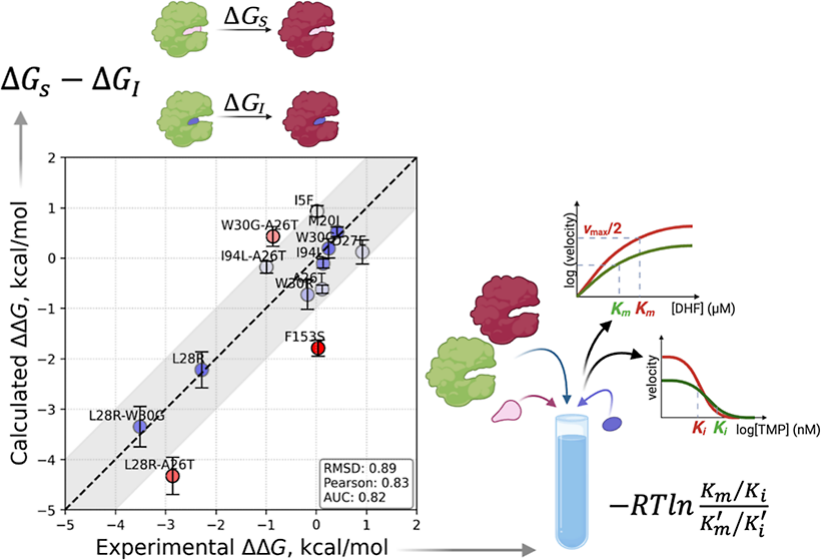

Understanding competitive inhibition at the molecular level is essential for unraveling the dynamics of enzyme–inhibitor interactions and predicting the evolutionary outcomes of resistance mutations. In this study, we present a framework linking competitive inhibition to alchemical free energy perturbation (FEP) calculations, focusing on *Escherichia coli* dihydrofolate reductase (DHFR) and its inhibition by trimethoprim (TMP). Using thermodynamic cycles, we relate experimentally measured binding constants (*K*_i_ and *K*_m_) to free energy differences associated with wild-type and mutant forms of DHFR with a mean error of 0.9 kcal/mol, providing insight into the molecular underpinnings of TMP resistance. Our findings highlight the importance of local conformational dynamics in competitive inhibition. Mutations in DHFR affect substrate and inhibitor binding affinities differently, influencing the fitness landscape under selective pressure from TMP. Our FEP simulations reveal that resistance mutations stabilize inhibitor-bound or substrate-bound states through specific structural and/or dynamical effects. The interplay of these effects showcases significant molecular-level epistasis in certain cases. The ability to separately assess substrate and inhibitor binding provides valuable insights, allowing for a more precise interpretation of mutation effects and epistatic interactions. Furthermore, we identify key challenges in FEP simulations, including convergence issues arising from charge-changing mutations and long-range allosteric effects. By integrating computational and experimental data, we provide an effective approach for predicting the functional impact of resistance mutations and their contributions to evolutionary fitness landscapes. These insights pave the way for constructing robust mutational scanning protocols and designing more effective therapeutic strategies against resistant bacterial strains.

## Introduction

Predicting competitive inhibition fates in point mutants of proteins is critical for the process of drug discovery, whereby one strives for inhibitors that are effective on a wide range of variants.^1^ This strategy would be critical for developing drugs which have efficacy within the time frames when resistance mutations arise and get permanently fixed.^2^ While accurate prediction of binding affinities helps prioritize compounds with the highest likelihood of success, reducing trial and error in drug discovery, experimental determination of binding affinities and development of suitable biochemical assays are expensive and time-intensive.^3^ Paradoxically, high failure rates in clinical trials often stem from poor understanding of binding affinities or off-target effects. One route to optimize these efforts is to develop computational approaches that allow virtual screening of compounds, identifying promising candidates early and reducing the costs of wet-lab experiments.

The level of accuracy and precision required for such predictions is currently provided by free energy perturbation (FEP) calculations.^4^ While accuracies below 0.5 kcal/mol are desired for reliable predictions, the current level of confidence provided by FEP calculations is around 1 kcal/mol.^5^ Improved accuracy over traditional methods such as docking or molecular mechanics force fields, which makes FEP more reliable in early stage drug design, stems from its full atomistic resolution, which enables modeling complex thermodynamic contributions like solvation and entropic effects.^6^ Conversely, simpler models often take such critical factors into consideration only as mean field effects, sacrificing accuracy and precision. FEP-based approaches are also adept at capturing small changes in molecular structure, such as substituent modifications and their impact on binding affinity. This feature is critical in lead optimization, where small structural modifications to drug candidates influence the activity.

While FEP calculations are costly compared to lower-resolution approaches, advancements in molecular dynamics (MD) simulations and GPU computing as well as fine-tuning of existing methods have made them ever more scalable and accessible.^7,8^ The ongoing development of FEP-based methods further ensures that these techniques keep pace with the increasing complexity of drug targets, allowing integration of large-scale FEP applications into pharmaceutical pipelines addressing real-life problems.^9^

Despite the many advances, constructing thermodynamic cycles that are suitable to make decisions on drug efficacy facing various mutations on the target is far from straightforward. Nevertheless, recent advances in alchemical free energy calculations have markedly improved our ability to predict how mutations affect inhibitor binding. For instance, by employing nonphysical transformation pathways within a thermodynamic cycle, it was possible to estimate changes in ligand binding affinities upon mutation with root-mean-squared errors (RMSEs) as low as 1.2 kcal/mol, providing a robust quantitative framework for protein design and drug resistance studies.^10^ This approach was extended to the challenging problem of kinase inhibitor resistance, comparing physics-based and data-driven approaches on Abl kinase mutations and showing that such techniques can accurately predict the impact of single-point mutations on drug binding.^11^ In the context of HIV-1 protease, alchemical free energy simulations were applied to a series of resistance-associated mutations, revealing that careful treatment of factors such as active site protonation is essential for consistent prediction of mutation-induced binding free energy changes.^12^ Furthermore, state-of-the-art protocols were shown to forecast resistance phenotypes in clinical Abl kinase mutants, achieving impressive classification accuracy and low RMSE values, underscoring the potential of these methods in precision medicine.^13^

Compared with transport or structural proteins, enzymes pose additional challenges due to the kinetic effects that need to be accounted for during lead optimization. In fact, the studies mentioned above do not explicitly incorporate the effect of the catalytic efficiency of the enzymes involved. Their primary focus is on quantifying changes in ligand binding free energies (ΔΔ*G*) upon mutation, which directly relate to inhibitor affinity rather than catalytic turnover. Integrating an explicit treatment of enzyme catalytic efficiency through the incorporation of *K*_m_ into a Bayesian framework was proposed to overcome this hurdle.^14^ This framework combines the computed free energy changes in the presence of the drugs with the experimental resistance factor data, which depend on both these free energy differences and *K*_m_, to more accurately predict how mutations alter resistance. The correction factor accounts for changes in catalytic activity that may accompany mutations, thereby linking the computed inhibitor binding energetics to the observed inhibition constants and ultimately resistance factor values. This enhancement, while probabilistic, provides a more robust thermodynamic framework that combines the dual effects of mutations on both ligand binding and enzyme kinetics, which is essential when mutations have a compensatory or antagonistic effect on catalytic turnover.

**Figure 1:**
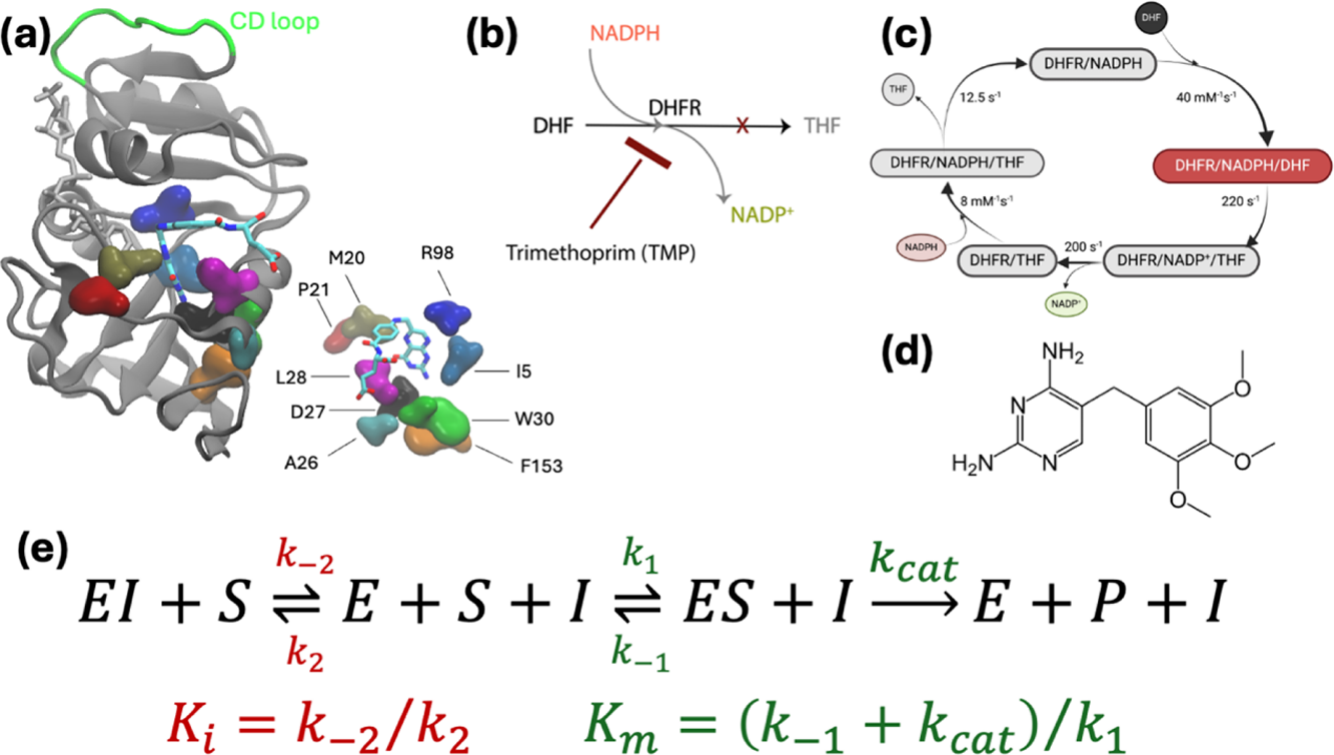
(a) DHFR structure displaying the cofactor NADPH (licorice representation colored gray). The substrate DHF and the residues most frequently observed in mutants arising in the morbidostat are also shown from a different perspective on the lower right. DHF is colored by atom type; the mutating residues are shown as volumetric blobs. The CD loop is marked in green; (b) illustration of the competitive inhibition with the hydride transfer step; (c) catalytic cycle of the enzyme with the simulated complex shown in red; (d) competitive inhibitor studied in this work, trimethoprim (TMP); (e) reaction scheme for competitive inhibition; *K*_i_ is the inhibitor’s dissociation constant, and *K*_m_ is the Michaelis constant.

In the current work, we propose an alternative framework that directly relates binding free energy differences to experimentally determined biochemical constants *K*_m_ and *K*_i_ measured under the evolutionary pressure of competitive inhibitors. We demonstrate the utility of this approach on the mutants of *Escherichia coli* dihydrofolate reductase (DHFR) (Figure 1a), which catalyzes dihydrofolate (DHF) to tetrahydrofolate (THF) (Figure 1b). We have chosen *E. coli* DHFR due to the extent of the evolutionary trajectories accumulated on this model system under the pressure from the competitor inhibitor trimethoprim (TMP) and the accompanying biochemical data for the emerging mutants.^15,16^ Moreover, the analyses of precatalytic conformers of DHFR mutants have enabled us and others to develop leads that steer the evolutionary trajectories away from the mutational pathways with extreme resistance to the drug,^17−19^ making it a testbed for developing drug optimization methodologies. We show that the thermodynamical cycle we propose is a good predictor of mutant fates in competitive inhibition and opens the way forward for high-throughput applications^20^ in predicting mutants arising under various evolutionary pressure scenarios.

Beyond single-site changes, we also examine double mutants in this system to illustrate how molecular-level epistasis can emerge from direct or indirect residue–residue interactions. Epistasis refers to the phenomenon in which the effect of one mutation depends on the presence of others and plays a pivotal role in shaping the ruggedness of adaptive fitness landscapes. While epistasis is often framed at the organismal level, focusing on overall fitness,^21,22^ our analysis targets the molecular dimension of epistasis,^23,24^ manifested as nonadditive changes in binding free energy, stability, or enzymatic activity. In this study, we specifically focus on whether combining two mutations produces binding energetics that deviate from the sum of their individual effects. We find that certain double mutants indeed exhibit substantial epistatic “rescue” or aggravation, highlighting how conformational rearrangements and altered residue contacts can drive nonadditivity. By elucidating these mechanistic underpinnings of epistasis, our framework broadens the understanding of how multiple mutations can impact enzyme–inhibitor interactions and guides the design of robust inhibitors resilient to complex evolutionary pathways.

## Methods

### System Preparation

All simulations for the systems are conducted with the NAMD program.^25^ The initial systems are based on the 1RX2 PDB-coded crystal structure^26^ where the cofactors NADPH and folate are bound to the enzyme. The TMP-bound systems are obtained by removing folate and docking TMP at the binding site. For both the DHF-bound and TMP-bound systems, each mutation is introduced with the VMD Mutator Plugin.^27^ Both Charmm22 with CMAP corrections and Charmm36 parameter sets for proteins are utilized.^28^ We report full results from the former in this article, but we verified that we have the same energy differences for the TMP-bound M20I, A26T, D27E, and I94L systems within error bars. DHF is simulated in a protonated form that models the precatalytic state; thus, the 5-protonated 7,8-dihydrofolate force-field parameters were used as reported in the literature.^29^ TMP is modeled in the protonated state as established in our previous work, where its force-field parameters are also listed.^30^ The water box is set to the dimensions of 65 × 87 × 65 Å with a padding of at least 10 Å TIP3P water layer in each direction of the protein. The salt concentration is set to isotonic conditions, 0.15 M, with K^+^ and Cl^+^ ions. Particle mesh Ewald summation is utilized to calculate long-range electrostatics with a cutoff distance of 12 Å and a switching distance of 10 Å. The RATTLE algorithm is applied to constrain bonds, and the Verlet algorithm is used with a time step of 2 fs. The temperature is controlled at 310 K by Langevin dynamics with a dampening coefficient of 5 ps^−1^. The pressure is set to 1 atm and regulated by the Langevin piston.

To obtain initial coordinates for FEP simulations, we conduct classical MD simulations on DHF-bound and TMP-bound WT systems. Due to convergence issues (see Results), equilibration simulations for the W30R and F153S mutants are also carried out. These systems are minimized for 10,000 steps. The resulting structures are subjected to 200 ns long production runs in the *NPT* ensemble, which we have shown in our previous work to be ample to equilibrate the mutants.^30,31^ In another study on DHFR, the last 2 ns of 10 ns long MD runs have been extracted to compute properties of the system.^19^ We therefore utilize various structures at the 10 ns time point and beyond, extracted from our 200 ns trajectories, as initial structures for the FEP simulations.

### Alchemical Free Energy Perturbation Calculations

The alchemical free energy perturbation method with Zwanzig’s formulation is followed for all the systems listed in Table 1.^32^ The implementation in the NAMD suite of programs is used.^33^ The best practices outlined by Mey et al.^5^ are applied. Force-field parameters and simulation conditions are the same as those in the previous subsection.

**Table 1:** Mutations for which FEP Calculations are Carried Out

system	no. of runs for Δ*G*_S_	no. of runs for Δ*G*_I_	notes
I5F	7	7	
M20I	4	4	
A26T	4	4	
D27E	4	7	
L28R^a^	6/6	4/4	
W30G	5	7	
W30R^a^	14/11	10/10	merge W30R & R30W simulations
I94L	4	4	
F153S	6	6	S153F simulations used for Δ*G*_S_
L28R-A26T	7	7	A26T mutation added to L28R
L28R-W30G	7	7	W30G mutation added to L28R
W30G-A26T	6	7	A26T mutation added to W30G
I94L-A26T	7	7	A26T mutation added to I94L

a Charge annihilation/creation runs are conducted separately and merged according to ref (34).

Systems selected from various equilibration MD simulation time points, which are at least 10 ns apart, are minimized, followed by 0.5 ns equilibration. We have tried λ = 16, 32, 64, and 128 for the TMP-bound L28R system and found the free energy differences to converge at λ = 32 windows, which we use for all systems presented in this work. Each window size is set to 200 ps, with the initial 50 ps being discarded for equilibration; repeating the calculations with 100 ps of equilibration and 100 ps of production yields similar results but with larger error bars. Hence, the production in each window is 150 ps long. The systems are run in both forward and backward directions.

To reduce singularities occurring during Leonard-Jones potential calculations, a soft-core potential is introduced at the middle λ. The charge-changing mutations are problematic because the results depend on the box size due to the long-range effects of electrostatic interactions. The protocol proposed by Morgan and Massi^34^ is used for charge correction, whereby one set of charge creation and another set of charge annihilation FEP runs are carried out. Since the two charge-changing mutations in this work are from a neutral side chain to a positively charged one (L28R and W30R), the created/annihilated charge is a chloride ion. The arithmetic average of the values obtained from these simulations provides the expected free energy difference for the mutation, free from box size effects, while the difference provides the free energy cost of charge creation in the water environment, Cl^−^ in this case. The convergence of the latter value across FEP runs provides an additional check for convergence of the free energy cost of the mutation.

Different initial structures are used in sampling to enhance the coverage of the potentially available conformational states. At least four different poses are chosen from the classical MD trajectory. These are from the 10, 50, 70, 100, 110, 150, 170, and 200 ns time points obtained from the trajectories described in the previous subsection and whose forward and backward simulations are completed in a stable manner. Our criterion for convergence is to have overlapping samples in all 32 windows for the forward and backward simulations when at least four FEP sets were merged in the Bennett-acceptance ratio (BAR) analyses. If additional sampling is necessary due to insufficient convergence of the free energy window overlaps, then more simulations selected from these time points are added to the pool. In two cases (L28R and F153S) where window overlaps and error bars did not yield consistent results, additional FEP simulations started from time points selected from the MD simulations of the mutated versions are conducted (see Results for details). Acquired data are merged and analyzed by the Bennett-acceptance ratio (BAR) method^35^ as implemented in the alchemlyb library.^36^ Errors are calculated from a simple RMSD averaging of the errors of the individual BAR calculations. All outputs and codes used in their analyses are provided on GitHub (see the Data Availability statement for details).

## Results

### Relating Competitive Inhibition to Alchemical Free Energy Calculations

The shifts in the conformational dynamics due to changes in substrate–enzyme interactions have recently been scrutinized for β-lactamase and underscore the effect of local dynamics on epistatic outcomes.^37^ It is therefore crucial to construct thermodynamic cycles suitable for the problem at hand whereby the reaction step(s) effective on local conformational dynamics are included in the calculations, while those that are independent of mutation types are assumed constant over the systems.

We will focus on the competitive inhibition of *E. coli* DHFR (Figure 1) by the inhibitor TMP. Ample biochemical experimental data have been published for this enzyme.^15^ DHFR converts dihydrofolate (DHF) to tetrahydrofolate (THF) by the transfer of one proton from the cofactor NADPH (Figure 1b) and another from the solvent environment. The catalytic cycle of *E. coli* DHFR consists of five steps (Figure 1c): Starting with the initial binding of the cofactor NADPH, the binding of DHF to the NADPH–enzyme complex to form a ternary complex follows. A hydride ion is then transferred from NADPH to DHF, reducing it to tetrahydrofolate (THF) and oxidizing NADPH to NADP^+^. Once the product THF is released from the enzyme, NADP^+^ dissociates, allowing it to bind new substrates. The hydride transfer step is rapid and not rate-limiting. Instead, the release of NADP^+^ is slower and often determines the overall rate of the catalytic cycle.^38^ The Met20 loop undergoes significant conformational shifts during the catalytic cycle.^26^ After hydride transfer, the loop must reopen to allow NADP^+^ to exit the active site. This conformational change is energetically demanding and slows the release of NADP^+^. In the precatalytic step, alignment for hydride transfer occurs. Precise positioning of NADPH and DHF allows for optimal orbital overlap necessary for the hydride transfer from NADPH to DHF. The enzyme’s conformational adjustments lower the activation energy by stabilizing the transition state, making the hydride transfer more efficient.^39−41^

Competitive inhibitors such as TMP (Figure 1d) target DHFR by mimicking the interactions of DHF at the active site with much higher affinity (nM vs. μM). Mutations affecting the precatalytic steps can alter binding affinities and conformational dynamics, leading to decreased inhibitor efficacy and drug resistance.^15,30^ The importance of the precatalytic step in DHFR underscores the intricate interplay among enzyme structure, dynamics, and function. By studying these early events in the catalytic cycle, we can better comprehend how enzymes achieve specificity and efficiency and how these processes can be manipulated for therapeutic purposes.

The reaction scheme for competitive inhibition is given in Figure 1e, with *K*_m_ being the Michaelis constant and *K*_i_ the inhibitor’s dissociation constant. Note that the latter is a purely thermodynamic quantity, whereas the former is kinetic since the product rate constant, *k*_cat_, contributes to this quantity. We define the free energy difference relating the relative *K*_m_/*K*_i_ values of the WT and the mutant protein, ΔΔ*G*_competition_
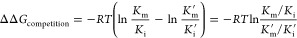
1for assessing the relative efficacy of the inhibitor in competitive inhibition. The ratio *K*_m_/*K*_i_ reflects how much more likely an enzyme is to bind to the substrate than the inhibitor, aiding in understanding the potency of the inhibitor in biochemical contexts. Similarly, the ratio / displays the binding propensity of a mutated enzyme toward the substrate relative to the inhibitor. The difference between these ratios quantifies the energetic advantage conferred by the mutation compared to that of the WT enzyme. In Table 2, we present the *K*_m_ and *K*_i_ data for *E. coli* DHFR for the WT enzyme and for those mutants that emerge when the bacteria are under evolutionary pressure from TMP. The nine single mutants that appear most frequently in morbidostat experiments are studied in this work;^15^ only P21L and R98P are left out since they involve changes from/to a proline residue for which FEP simulations are currently not feasible due to the ring structure fused with the protein backbone. We also include four of their double mutant combinations for which biochemical data are available,^15^ leading to a test set of 13 mutants.

**Table 2:** Biochemical Data (*K*_m_ and *K*_i_) and the ΔΔ*G*_competition_ Values Calculated via eq 1 for the Mutants Studied

mutants	*K*_m_ (μM)^a^	*K*_i_ (nM)^a^	ΔΔ*G* (kcal/mol)
**WT**	**2.86**	**4.59**	
I5F	7.68	11.90	0.02
M20I	3.57	2.90	0.42
A26T	7.65	10.10	0.12
D27E	56.40	20.21	0.92
L28R	0.95	61.94	−2.28
W30G	9.49	10.20	0.25
W30R	4.97	10.53	−0.17
I94L	14.87	19.15	0.14
F153S	11.32	17.00	0.04
L28R-A26T	1.87	309.07	−2.86
L28R-W30G	0.65	309.00	−3.51
W30G-A26T	14.87	96.77	−0.86
I94L-A26T	11.05	88.44	−0.99

a Data reported in ref (15).

Table 2 underscores the fact that, while antibiotic resistance through target modifications is often associated with decreased drug and substrate affinities caused by mutations, experimental measurements suggest the presence of additional resistance mechanisms. Moreover, *K*_i_ values alone are insufficient to fully explain the TMP resistance. Within the bacterial cell, various other factors including DHFR abundance, catalytic efficiency, thermal stability, nutrient and metabolite availability, excess DHF accumulation, and the demand for THF play significant roles in determining bacterial fitness in the presence of TMP. Of the mutants listed in Table 2, those involving L28R and W30R are the most frequently observed in the morbidostat as the first replacement in the coding region,^15,31^ consistent with the listed ΔΔ*G*_competition_ values calculated by applying eq 1. Moreover, multiple mutants containing the L28R mutation are among the toughest to eradicate by drugs,^18^ confirming the further lowered values as listed in Table 2.

We next seek a thermodynamic cycle that will be useful for the prediction of the quantity in eq 1. Under the steady-state assumption, which is valid when product formation is linear in time, we arrive at the relation for the concentration of inhibitor-bound enzyme
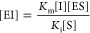
2

**Figure 2:**
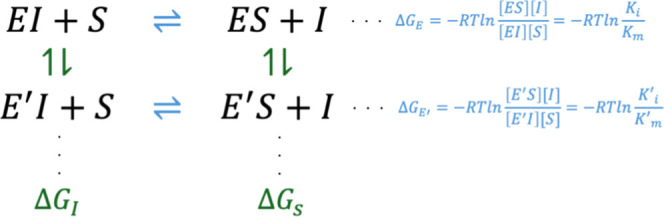
Thermodynamic scheme depicting a cycle for which the alchemical FEP simulations are feasible for the vertical (green) reactions, while the substrate–inhibitor exchange reactions occurring in physical reality are shown in the horizontal (blue) reactions. Note that the top horizontal reaction is the 1st and 3rd steps of the competitive inhibition scheme in Figure 1e. The last equalities in the blue equations follow from eq 2.

Our aim is to use classical force fields for an enzyme to predict the relative effect of point mutations on the evolutionary outcomes of its overall function. We therefore focus on the equilibrium between the inhibitor-bound enzyme (EI) and the substrate-bound enzyme (ES). Writing this equilibrium situation twice, once for the WT enzyme (E) and once for the mutant (E′), we arrive at the thermodynamic cycle depicted in Figure 2; the detailed derivation of this cycle relies on rapid equilibrium and initial rate conditions; the full derivation and the assumptions involved are included in the Supporting Information. The horizontal equilibria are for the competition of the substrate and the inhibitor for the same binding site in enzyme E, Δ*G*_E_, and mutant enzyme E′, . The vertical equilibria represent the cost of mutation in the inhibitor-bound enzyme, Δ*G*_I_, and the substrate-bound enzyme in the precatalytic step, Δ*G*_s_. It is these vertical values that may be obtained via FEP simulations.

The sum over the free energy differences around the cycle is zero
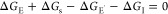
3

Rearranging and substituting eq 2
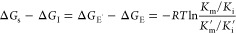
4

Equation 4 is our final expression linking the FEP simulations to biochemical experimental results, which we defined in eq 1. We use FEP simulations to introduce a point mutation to the WT enzyme. We carry out FEP simulations on both the substrate-bound (ES) and the inhibitor-bound (EI) forms of the enzyme. The difference in the free energies is expected to provide the right-hand side, which is obtained from experimentally measured quantities. Our main assumption is that the mutations make a difference predominantly at the precatalytic step when the enzyme samples conformations that are best suited for the catalysis to take place,^42−44^ while the product release is not dependent on the mutations on the enzyme but enters the final observed reaction rates as a common multiple in all mutants, bridging the time scale difference.^45^ Thus, our assumption is that the mutations do not affect the catalytic efficiency of the enzyme and so *K*_m_ and *K*_i_ are considered to reflect the binding of the substrate and inhibitor, respectively. Therefore, their ratio can be used to estimate the difference in binding. This reduces the problem to that of two alternative ligands binding to a given protein, similar to other standard applications of FEP approaches. We will show that this assumption holds for the system we study in this work and might hold for other cases, although its effectiveness should be assessed for the enzyme at hand in future applications.

### DHFR Mutation Fates Predicted by FEP Simulations

**Figure 3:**
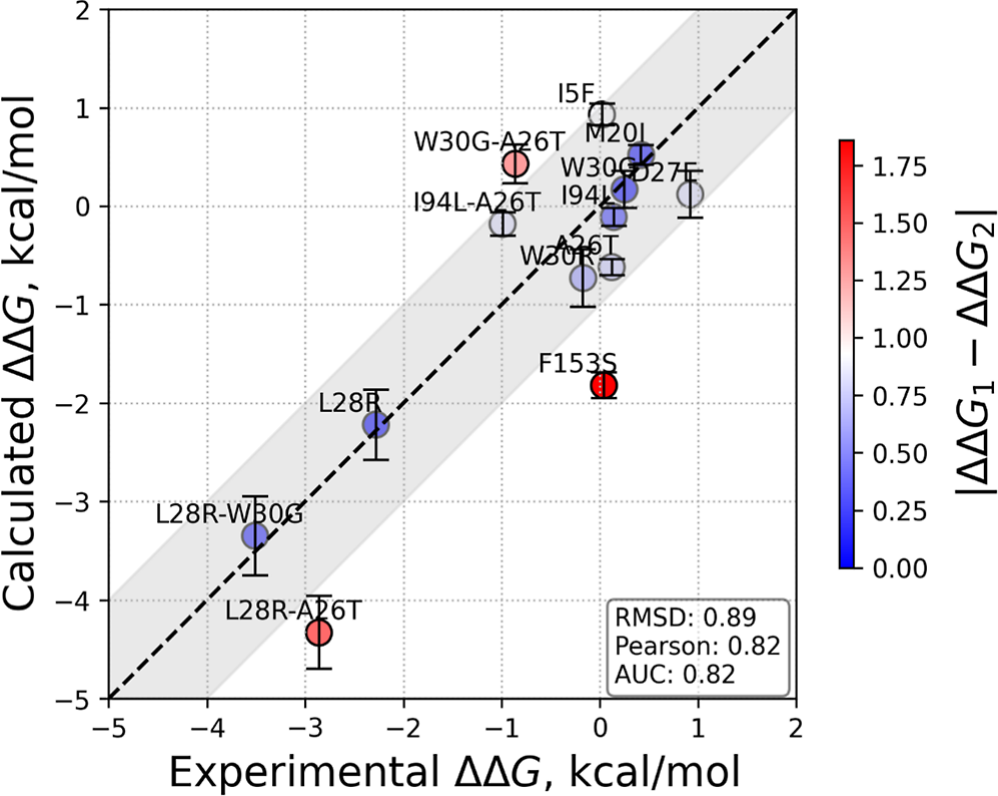
Calculated vs experimental average ΔΔ*G* values from Tables 2 and 3 (*x*- and *y*-axes, respectively) with the ±1 kcal/mol error margin highlighted in gray. The error bars are those values listed in the last column of Table 3. The data points are colored from blue (less deviation) to red (more deviation) according to their departure from the *y* = *x* line. The measures of deviations in terms of RMSD of the data, Pearson correlation coefficient, and AUC are provided in the inset.

The calculated values for our case study of competitive inhibition of *E. coli* DHFR by TMP are listed in Table 3. We note that the experimental data of Table 2 were collected under the conditions required for the application of eq 4. The calculated data are visualized against the experimental counterpart values in Figure 3. The performance of the predicted values is quantified using three metrics: To evaluate the overall agreement between predicted and experimental ΔΔ*G* values, we calculated the root-mean-square deviation (RMSD) of the differences between the experimental and predicted values. To assess the linear correlation between the predicted and experimental ΔΔ*G* values, we computed the Pearson correlation coefficient. Finally, to assess the discriminative ability of the model, we computed the area under the receiver operating characteristic curve (AUC) by thresholding experimental ΔΔ*G* values at 0 kcal/mol to define binary labels. Predicted ΔΔ*G* values were used as continuous scores, and the AUC was calculated using the trapezoidal rule. Our findings point to the applicability of our approach with high accuracy and further disclose the mechanisms that lead to these fates.

**Table 3:** Calculated FEP Values^a^

system	Δ*G*_S_ (kcal/mol)	Δ*G*_I_ (kcal/mol)	ΔΔ*G* (kcal/mol)
I5F	−0.66 ± 0.08	−1.59 ± 0.08	0.93 ± 0.11
M20I	7.94 ± 0.07	7.42 ± 0.07	0.52 ± 0.10
A26T	−1.90 ± 0.07	−1.28 ± 0.07	−0.62 ± 0.10
D27E	10.02 ± 0.16	9.91 ± 0.18	0.12 ± 0.24
L28R	−45.62 ± 0.62	−43.40 ± 0.26	−2.22 ± 0.36
W30G	4.96 ± 0.15	4.79 ± 0.11	0.17 ± 0.19
W30R	−38.81 ± 0.21	−38.09 ± 0.20	−0.73 ± 0.29
I94L	−3.97 ± 0.06	−3.86 ± 0.07	−0.11 ± 0.09
F153S	−4.22 ± 0.09	−2.40 ± 0.09	−1.82 ± 0.13
L28R-A26T	−48.27 ± 0.26	−43.94 ± 0.26	−4.33 ± 0.37
L28R-W30G	−41.68 ± 0.28	−38.34 ± 0.28	−3.35 ± 0.40
W30G-A26T	0.15 ± 0.16	−0.28 ± 0.12	0.43 ± 0.20
I94L-A26T	−5.76 ± 0.08	−5.58 ± 0.09	−0.18 ± 0.12

a Δ*G*_S_ and Δ*G*_I_ are the direct outputs of the related FEP calculations; the final ΔΔ*G* is calculated via the left-hand side of eq 4.

Inspection of the experimental data in Table 2 shows that, according to eq 1, there is only one mutation that displays significant stabilization of the mutant while in competition with TMP. This is L28R, which has a ΔΔ*G*_competition_ of −2.28 kcal/mol. The double mutants L28R-A26T and L28R-W30G are further stabilized, along with W30G-A26T and I94L-A26T, which are slightly stabilized by ca. 1 kcal/mol. We find that the mutants destabilize the inhibitor in all but one case (M20I), as one would expect in a resistance mutation, i.e., they display larger *K*_i_ than the WT. However, in all single mutants except L28R, the substrate-bound complex ES is also destabilized with respect to the WT. The latter is an expected outcome since the competitive inhibitor targets the same modified binding site. The net effect is a ΔΔ*G*_competition_ value within 1 kcal/mol of the WT, and this may be the main reason why these usually appear as second or later mutations in morbidostat trajectories. The surviving mutants are then those losing less binding affinity for the substrate than to the inhibitor.

One may follow this outcome from the FEP results with a protein-centric perspective rather than the binder-centric perspective of the experiments. The cost of a mutation may be positive or negative in the presence of the substrate or the inhibitor, as shown by the range of values taken on by Δ*G*_S_ and Δ*G*_I_ (Table 3). The charge-introducing mutations are stabilizing for the protein in the presence of both DHF and TMP (e.g., L28R and W30R), while charge-maintaining ones may be positive or negative, depending on the local environment and how the dynamics are altered. Nevertheless, the effect of the mutation is always in the same direction for the DHF- and TMP-bound forms, and the final stabilization is decided upon by the relative effect of these two values.

### Interpreting Epistasis for Individual Binders and the Overall Outcomes from a Molecular Perspective

There is ongoing discussion in the literature regarding the extent to which protein sequence–function relationships are governed by pairwise interactions and higher-order effects.^46,47^ The answer to this question is important because it determines the ease with which evolutionary landscapes may be constructed, both experimentally and computationally. Interpreting fitness landscapes requires integrating molecular-level interactions, mutation effects, and broader evolutionary dynamics. While free energy differences due to changes in atomic-level interactions are far from being the only contributors to the fitness values, they are certainly among the most important.^48^

In our study, the double mutants under investigation occur in residues that are in close proximity to each other or interact directly with the substrate or inhibitor. Consequently, nonadditivity in the measured free energy changes is expected. Our analysis quantifies the degree of nonadditivity by comparing the observed free energy differences of double mutants with the sum of the effects of the corresponding single mutants (Table 4). It is important to emphasize that our focus is on understanding how direct or indirect interactions between these residues contribute to deviations from simple additivity. While our discussion is framed at the molecular level, such insights are essential for elucidating one of the important components underlying the evolutionary drivers of epistasis. We do not claim that our molecular analysis alone explains epistasis; rather, it provides a detailed view of how local interactions shape the free energy landscape, which, in turn, can contribute to the evolutionary dynamics observed at the organismal level.

**Table 4:** Role of Epistasis for Individual Binding Events of the Substrate and Inhibitor^a^

mutants	Δ*G*_S_ (kcal/mol)	Δ*G*_S_^e^ (kcal/mol)	epistasis in substrate-bound DHFR	Δ*G*_I_ (kcal/mol)	Δ*G*_I_^e^ (kcal/mol)	epistasis in inhibitor-bound DHFR
L28R-A26T	−48.27 ± 0.26	−47.52 ± 0.62	−0.75 ± 0.68	−43.94 ± 0.26	−44.68 ± 0.27	0.74 ± 0.37
L28R-W30G	−41.68 ± 0.28	−40.66 ± 0.64	−1.02 ± 0.70	−38.34 ± 0.28	−38.61 ± 0.28	0.27 ± 0.40
**W30G-A26T**	0.15 ± 0.16	3.06 ± 0.17	**−2.91±0.23**	−0.28 ± 0.12	3.51 ± 0.13	**−3.79±0.18**
I94L-A26T	−5.76 ± 0.08	−5.87 ± 0.09	0.11 ± 0.12	−5.58 ± 0.09	−5.14 ± 0.10	−0.44 ± 0.13

a Δ*G*_S_ and Δ*G*_I_ are the direct outputs of the related FEP calculations, while their counterparts with the superscript *e* indicate the expected free energy difference if the individual mutations were additive. Their difference indicates the extent of epistasis, and those that deviate by larger than the usual tolerance of 1 kcal/more are shown in bold.

**Figure 4:**
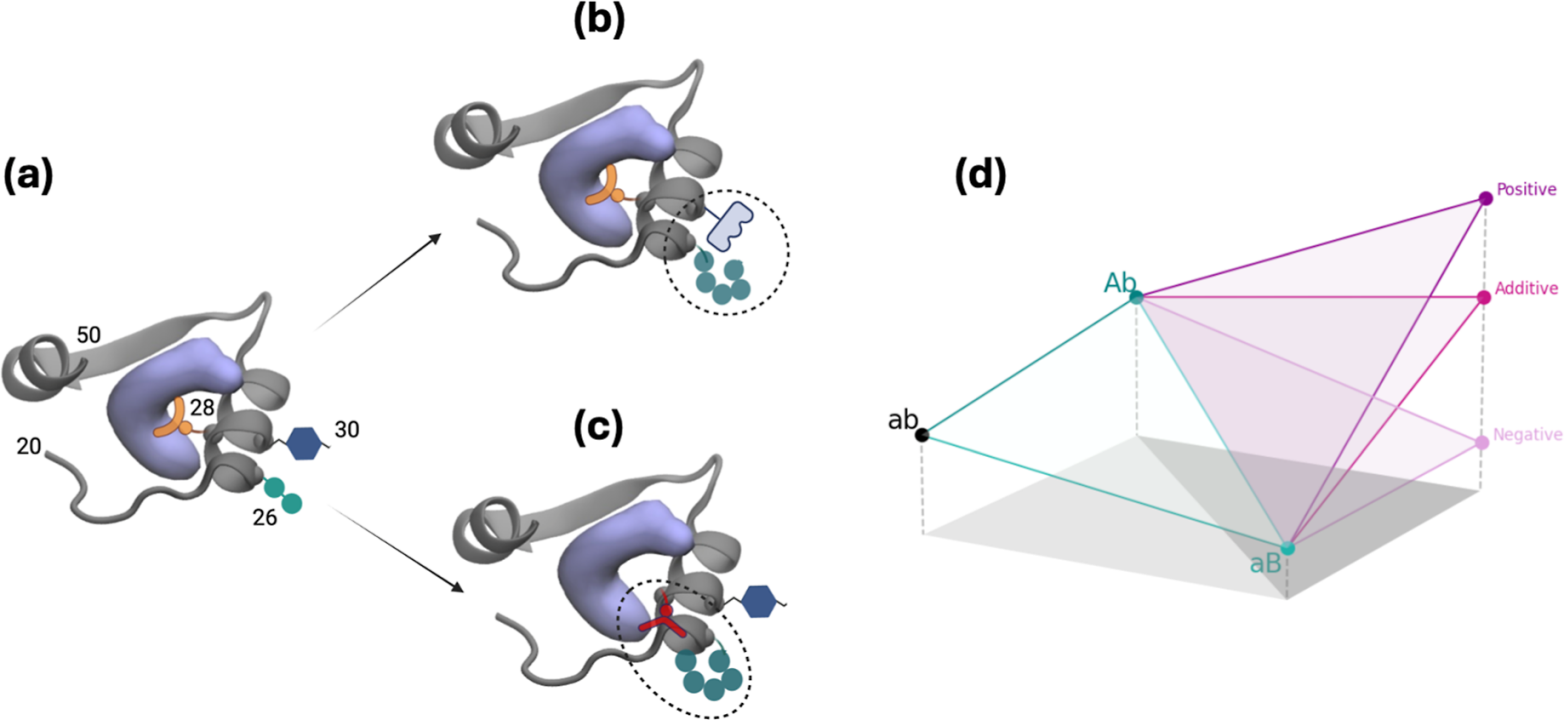
Schematic illustration of the effects of frequent mutations observed near the substrate binding site of DHFR for (a) WT, (b) double mutation at positions 26/30, and (c) double mutations at positions 26/28. Direct or indirect interactions of the side chains with each other and/or the substrate may be responsible for epistasis. The protein structure (PDB code 1rx2) for residues 20−50 is shown in cartoon, the substrate DHF is shown as a volumetric purple blob, and the positioning of the side chains of residues 26, 28, and 30 are illustrated by various geometric shapes. (d) Fitness assessment where larger values correspond to lower free energy difference; capital letters imply point mutations. Combining mutations can result in additivity (i.e., no epistasis), as exemplified by the I94L-A26T double mutation. An increase over the expected fitness, e.g., for the DHF-bound L28R-A26T system, leads to positive epistasis, and negative epistasis is exemplified by TMP binding to the same system. (a)−(c) were created with BioRender.com.

We compare the calculated versus the expected Δ*G*_S_ and Δ*G*_I_ values, where the expected values are obtained by the simple summation of the individual free energy differences of the single mutants (Table 3). For example, in the case of the W30G-A26T double mutant, the W30G mutation contributes a positive free energy difference of 4.96 kcal/mol for DHF binding (and 4.79 kcal/mol for TMP binding), while A26T contributes a negative difference of −1.90 kcal/mol for DHF (and −1.26 kcal/mol for TMP). In the absence of nonadditive effects, one would expect the double mutant to have a ΔΔ*G* of approximately 3.06 kcal/mol (i.e., 4.96 + (−1.90)) for DHF binding. However, our simulations yield an actual value of only 0.15 kcal/mol for DHF binding and −0.28 kcal/mol for TMP binding, indicating a significant nonadditivity. We interpret this “epistatic rescue” (calculated as the difference between the expected and observed ΔΔ*G* values, amounting to approximately −2.91 kcal/mol for DHF and −3.79 kcal/mol for TMP) as arising primarily from conformational adjustments in the protein, rather than from direct interactions with the binder. This interpretation is illustrated in Figure 4a, showing how the spatial arrangement of the A26 and W30 side chains may compensate for each other’s destabilizing effects. Importantly, while our discussion quantifies these nonadditive effects at the molecular level, we do not imply that free energy can be rigorously decomposed into isolated contributions; rather, we use these trends to provide qualitative insights into how specific local interactions deviate from additivity. In contrast, the double mutants involving I94L and A26T, which lack direct or indirect cross-interactions, show negligible epistasis (Tables 4 and 5).

**Table 5:** Role of Epistasis in Competitive Inhibition^a^

mutants	ΔΔ*G* (kcal/mol)	ΔΔ*G*^e^ (kcal/mol)	apparent epistasis (kcal/mol)
**L28R-A26T**	−4.33 ± 0.37	−2.84 ± 0.37	**−1.49±0.53**
**L28R-W30G**	−3.35 ± 0.40	−2.05 ± 0.41	**−1.30±0.57**
W30G-A26T	0.43 ± 0.20	−0.45 ± 0.21	0.88 ± 0.27
I94L-A26T	−0.18 ± 0.12	−0.73 ± 0.38	0.55 ± 0.40

a ΔΔ*G* is calculated via the left-hand side of eq 4, while the superscript *e* indicates the expected free energy difference if the individual mutations were additive. Their difference is labeled 'apparent epistasis' as discussed in the text.

Different outcomes are observed for the double mutants involving L28R. For the DHF-bound forms, these mutants exhibit an epistasis on the order of −1 kcal/mol, whereas for the TMP-bound forms, the epistasis is positive. In the case of DHF-bound L28R-A26T (Figure 4b), the A26T mutation appears to modify the direct interaction of the R28 side chain with the ligand. Although the individual contributions to ΔΔ*G* are small, the opposing effects for DHF and TMP binding lead to a notable net nonadditivity. As the inhibitor is smaller than the substrate (molar mass 290 and 441 g/mol, respectively; also see Figure 1a,d), thereby establishing fewer contacts with the enzyme, we might in general expect such differentiated epistatic effects for mutations that directly affect the binding site. Here, as in the previous example, our analysis is intended to highlight qualitative deviations from additivity that reflect local structural rearrangements, rather than to claim a rigorous partitioning of the free energy into independent components.

### Some Comments on Achieving Convergence

Accurately exploring the necessary conformational space for each alchemical state is vital to the success of these results.^49^ However, this becomes problematic when a significant conformational rearrangement is required to transition from the reference to the target binder–receptor complex. While extending simulation times and employing enhanced sampling strategies can help, their impact is varied and especially difficult when the free energy barriers between conformational states are high or when adopting and maintaining the desired target conformation proves challenging. In Table 1, we have listed the number of simulations required to get converged free energy values for either the DHF-bound or TMP-bound forms. Our criterion for convergence was to have overlapping samples in all 32 windows for the forward and backward simulations when at least four FEP sets were merged in the BAR analyses. For some of these cases, we have achieved such convergence in four sets, but we have had to increase the simulation sets up to 7 in many cases. Overlaps in windows are exemplified in Figure S1. The full set of outputs is also provided (please see the Data Availability statement for details).

Two of the mutations we have handled are charge-changing: L28R and W30R. A recent systematic study has shown that charge-changing mutations are particularly prone to convergence issues, while usual FEP protocols are adept at predicting consistent free energy costs of neutral side chain replacements.^20^ The significance of charge corrections due to electrostatic contributions to binding emerged due to Ewald summation requirements in periodic boundaries. We have used the protocol from the literature^34^ as described under the Methods section, which greatly alleviated charge correction errors. This approach also provides an additional check on the convergence of the free energy differences if the cost of the Cl^−^ creation in water is converged to a value of 83.0 ± 0.5 kcal/mol in this case. On the other hand, the calculated BAR errors are larger due to merging two simulations to predict the free energy change.

**Figure 5:**
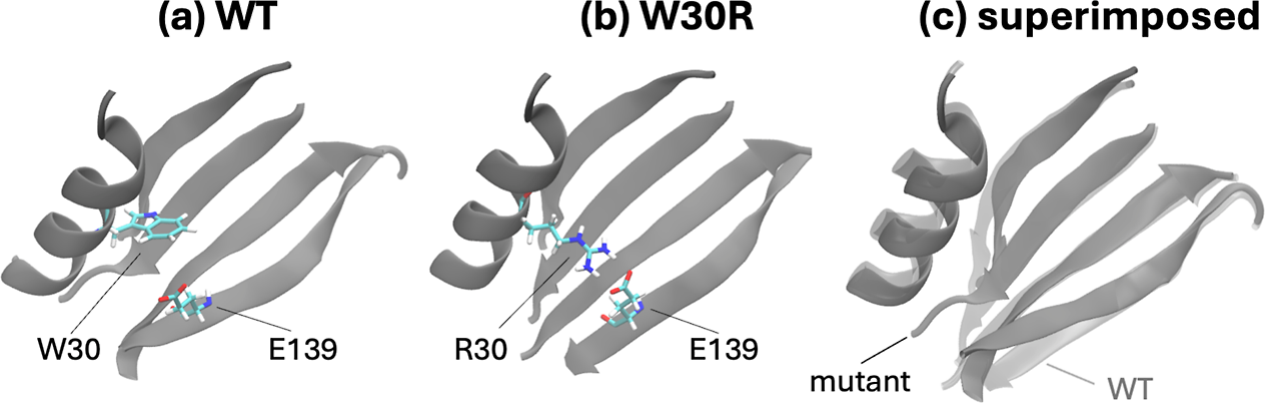
Relative positioning of residues 30 and 139 in the classical MD simulations of (a) WT and (b) W30R mutant. (c) The salt bridge established in the mutant “pulls” the α-helix and β-sheet where these residues reside toward each other. The trajectories display a maximum 2 Å RMSD for the total protein.

We had additional challenges in simulating the W30R mutation. We have shown in our previous work that the effect of the W30R change is due to the salt bridge established between the newly created R30 side chain and E139 on the β-sheet residing on the opposite side, further away from the binding region (compare Figure 5a,b).^15^ The separation between the two residues is thus greatly reduced with a salt bridge distance at a baseline value of 2 Å, which samples values up to 7 Å during a 200 ns trajectory. It also leads to an overall shrinkage in the distance between the α-helix accommodating R30 and the β-sheet where E139 resides (Figure 5c). This situation is hardly reproducible during a FEP simulation due to the gradual onset of the interactions and the relatively short simulation times that may not allow for sampling all of the possible interaction scenarios. We have therefore included a second set of simulations where we carried out FEP simulations for the R30W mutations, starting from the salt-bridged orientations and using the negative value of the final free energy differences. Only when we have combined the two sets of simulations have we been able to converge the overlaps in the λ windows along with the converged ion creation value as described for L28R.

Finally, the F153S mutation has proven to be another hard case. This residue is located near the C-terminus and is distal from the binding site. In fact, there is no apparent effect of the mutation that may be captured by comparing 200 ns long classical MD simulations for WT and F153S mutants via visual inspection of the trajectories. However, our previous work, which followed large shifts in hydrogen bond occupancies, has shown that this mutation has an allosteric effect on DHFR, interfering with the motions of the CD loop (Figure 1a) and revealing a cryptic site that is more than 30 Å away.^31^ As in the previous case of W30R, such long-range effects may not be captured by the gradual changes introduced into the side chains during the relatively short FEP simulations. Moreover, the overlaps in the first window of the reverse runs were very poor (Figure S1a) in the F153S simulations of the DHF-bound systems. Conversely, the S153F systems had well-behaved overlaps (Figure S1b), and we have reported the negative of the value predicted for these systems in Table 3. We did not have this problem for the TMP-bound system, hinting that allosteric communication requires DHF.

For the double mutants, we have used the systems we have studied extensively in our earlier work as the base:^15,30^ L28R for the L28R-A26T/W30G mutations, W30G for the W30G-A26T change, and I94L for the I94L-A26T change. We used the time points selected from the 200 ns MD simulations of the system with the single mutation to add the second mutation. We report the values in Table 3 as the sum of the costs of the two FEP simulations. However, we have also tried introducing both mutations at the same time for the I94L-A26T change. We find that it is not straightforward to converge simultaneous mutations even for this case, where both single mutants gave converged results in just four FEP simulations.

We acknowledge that while our enhanced convergence criteria for specific mutations were necessary to overcome known sampling challenges, they may introduce systematic differences compared to cases in which less sampling was required. We further note that the possibility that convergence in some systems might be partly fortuitous cannot be entirely excluded and warrants additional validation in future studies. Some strategies that might be employed in future work to systematically refine and further validate our methodology include increasing the number of replicates under carefully selected conditions to further reduce stochastic error and evaluate the reproducibility. In addition, one can test alternative sampling strategies, such as parallel tempering or replica exchange, to more thoroughly explore conformational space to select initial structures for FEP simulations, particularly for challenging mutations and in analyzing the effects of introducing double mutations.

## Conclusions

The development of FEP-based methods addresses critical challenges in drug design including accuracy, efficiency, and cost-effectiveness. By refining these methods, faster and more precise drug discovery processes may be achieved, ultimately improving patient outcomes and reducing development costs. FEP can model the interactions of drugs with mutated or polymorphic targets, aiding in the development of personalized therapeutics. For diseases with genetic variations, such as cancer or antibiotic resistance, FEP holds immense potential in the design of tailored drugs. For high-throughput predictions, well-established computation protocols will prove essential.^20^

In this article, we utilize a thermodynamic cycle that informs on the relative efficiency of an enzyme in the presence of a competitive inhibitor and relate it to the biochemical constants *K*_m_ and *K*_i_. Our framework is built upon the key assumption that under steady-state conditions, the Michaelis constant (*K*_m_) largely reflects substrate binding affinity (approximating *K*_d_) because in *E. coli* DHFR, the rate-determining step is the product release, which remains largely unaltered by mutations affecting the binding site. Second, we assume that kinetic contributions beyond binding (e.g., those from *k*_cat_) can be decoupled from the binding process such that differences in observed inhibition concentrations predominantly stem from alterations in binding free energy. We note that if mutations were to affect catalytic turnover or product release significantly, these assumptions might break down. However, for the DHFR system under competitive inhibition by trimethoprim (TMP), experimental evidence indicates that changes in binding affinity dominate the resistance phenotype. This assumption thereby reduces our problem to that of comparing binding free energies of two competitive ligands (substrate and inhibitor), a standard scenario in alchemical free energy calculations.

We demonstrate the applicability of this approach on 13 variants of *E. coli* DHFR that arise in bacterial colonies under the intense evolutionary pressure of TMP. Our thermodynamical cycle is based on FEP simulations carried out on substrate-bound protein in the precatalytic conformation, which has been argued to be the step most significantly affected by the mutations in enzymes. This is because, while mutations often shift population states of the enzyme, favoring catalytically competent conformations, these shifts do not necessarily extend to altering the pathways for product release, especially when product egress mechanisms rely on conserved structural features.^50^ This assumption holds well for our predictions, with 11 out of the 13 systems studied falling within the ±1 kcal/mol range (Figure 3), barring F153S and L28R-A26T, which are in the ±2 kcal/mol range. The measures of the overall deviations from experimental values, in terms of RMSD of the data (0.89), Pearson correlation coefficient (0.83), and AUC (0.82), are all favorable, considering the complexities of the subtle conformational changes and gradual ligand rearrangements. The largest discrepancy is for the F153S mutant, whereby our simulations predict a negative ΔΔ*G*_competition_ value in favor of the substrate, while experimental data finds the difference negligible. We note that F153S is an allosteric resistance-conferring mutation for which other mechanisms that come into play may not have been captured within the time frame of our simulations. Thus, the nuanced effects of mutations on protein behavior pose difficulties in predicting free energy changes; e.g., challenges emerge when compensating for charge changes, necessitating usage of additional runs or simulation of the reverse mutation to cover the possible conformational range.

Our approach introduces a protein-centric view of the competitive inhibition mechanisms and allows us to interpret the effect of the mutations on the substrate-bound and the inhibitor-bound forms of the enzyme separately. Thus, substrate binding and inhibitor binding may be assessed individually, which is a big advantage for rational drug design purposes. In particular, displaying similar free energy differences for both binders implies that the effect of the mutation does not directly involve the binding site. Moreover, epistasis may also be interpreted in a novel way, and background mutation selection for high-throughput mutational scanning experiments may be suggested based on this additional structural information. With the currently available computational resources, carrying out a total mutational scan on the WT, followed by mutational scans in the background of mutations selected based on this detailed knowledge, has become possible.^20^ This capability opens the way forward for interpreting epistatic effects on fitness landscapes.

## Data Availability

All automated scripts for calculation setup and the outputs of the FEP simulations can be found at https://github.com/midstlab/cyclescript.

## Supporting Information Available

Text on the derivation of the thermodynamic cycle of Figure 2 and probability densities for the energy distributions in DHF-bound F153S and S153F runs (PDF)
