# Supplementary material for: A Thermodynamic Cycle to Predict the Competitive Inhibition Outcomes of an Evolving Enzyme
Source: J Chem Theory Comput. 2025 Apr 23;21(9):4910–20. doi: 10.1021/acs.jctc.5c00193 (PMC12080111; doi:10.1021/acs.jctc.5c00193)

Supporting Information for

A thermodynamic cycle to predict the competitive inhibition outcomes of an evolving enzyme

Ebru Cetin [a†], Haleh Abdizadeh [a‡], Ali Rana Atilgan [a], Canan Atilgan [a*]

[a] Faculty of Engineering and Natural Sciences, Sabanci University, 34956 Istanbul, Türkiye
* Email: canan@sabanciuniv.edu

Present addresses:
† Department of Chemistry & Biochemistry, University of Arizona, Tucson, Arizona 85721, United States
‡ Department of Strategic Development, University of Twente, 7500 AE Enschede, The Netherlands

Derivation of the thermodynamic cycle of Figure 2

Simple Michaelis-Menten kinetics where only one ligand binds to the enzyme are described by,

$$E + S \underset{k_r}{\overset{k_f}{\rightleftharpoons}} ES \overset{k_{cat}}{\longrightarrow} E + P \tag{S1}$$

However, in competitive binding two ligands compete for the same binding site, and competitive inhibition kinetics are described by,

$$EI + S \underset{k_2}{\overset{k_{-2}}{\rightleftharpoons}} E + S + I \underset{k_{-1}}{\overset{k_1}{\rightleftharpoons}} ES + I \overset{k_{cat}}{\longrightarrow} E + P + I \tag{S2}$$

Total enzyme concentration is $[E]_0 = [E] + [ES] + [EI]$ with $[E]_0$ being . When the steady-state assumption is imposed, corresponding rates for the different enzyme compounds become,

$$\frac{d[E]}{dt} = 0 = -k_1[E][S] + k_{-1}[ES] + k_{cat}[ES] - k_2[E][I] + k_{-2}[EI]$$
$$\frac{d[ES]}{dt} = 0 = k_1[E][S] - k_{-1}[ES] - k_{cat}[ES] \tag{S3}$$
$$\frac{d[EI]}{dt} = 0 = k_2[E][I] - k_{-2}[EI]$$

and the product formation rate is measured under conditions in which the substrate and inhibitor concentrations, $[S]$ and $[I]$ do not change substantially, and an insignificant amount of product has accumulated. Defining $K_i = k_{-2}/k_2$ and $K_m = (k_{-1} + k_{cat})/k_1$ and manipulating the foregoing equations, the inhibitor-bound enzyme concentration is found to depend on $K_m$and K_i as follows:

$$[EI] = \frac{K_m[I][ES]}{K_i[S]} \tag{S4}$$

Similarly, for the mutant enzyme E' we have the relation,

$$[E'I] = \frac{K_m[I][E'S]}{K_i[S]} \tag{S5}$$

If two main assumptions are met, we can isolate the equilibrium between the inhibitor bound enzyme (EI) and substrate bound enzyme (ES) from equation S2 and write the reaction sequence as follows:

$$EI + S \rightleftharpoons ES + I \tag{S6}$$

These are, **(i)** rapid equilibrium assumption for binding whereby the intermediate enzyme–substrate–inhibitor states are formed and broken down quickly relative to product formation, so that these binding steps remain near equilibrium on the timescale of catalysis. In other words, the binding of the enzyme to the substrate and the inhibitor is fast compared to any subsequent steps, so one can treat it as a single reversible reaction; and **(ii)** there is negligible product accumulation which is valid at initial-rate conditions whereby

measurements are taken under conditions of the conversion of substrate to product (the catalytic step) is slow enough or substrate is in sufficient excess that product does not significantly accumulate. This ensures focusing on how S and I compete for enzyme binding.

Writing equation S6 twice, once for the WT enzyme (E) and once for the mutant (E') we arrive at the thermodynamic cycle depicted in Figure 2:

$$
\begin{aligned}
EI + S &\rightleftharpoons ES + I \qquad\qquad \Delta G_E \\
E'I + S &\rightleftharpoons E'S + I \qquad\qquad \Delta G_{E\prime}
\end{aligned}
\tag{S7}
$$

with ΔG_E and $\Delta G_{E\prime}$ being the free energy differences for the exchange of ligand in the WT and mutant enzymes, respectively. The free energy differences in the horizontal equilibria are given by,

$$
\begin{aligned}
\Delta G_E &= -RTln\frac{[ES][I]}{[EI][S]} = -RTln\frac{K_i}{K_m} \\
\Delta G_{E\prime} &= -RTln\frac{[E'S][I]}{[E'I][S]} = -RTln\frac{K'_i}{K'_m}
\end{aligned}
\tag{S8}
$$

where the final equalities arise from substituting equations S4 and S5 into equations S8.

Next, let us call the vertical free energy changes calculated from free energy perturbation simulations ΔG_I and ΔG_S for the inhibitor and substrate bound forms, respectively. Note that the FEP simulations do not explicitly have free substrate or free inhibitor for the respective cases; and since there is one molecule of each in simulation boxes of the same size, the additive constant for standardizing the free energy differences will cancel out in the $\Delta\Delta G$ terms.

Finally, since the free energy differences around the thermodynamic cycle provided in equation S7 must sum to zero,

$$
\begin{aligned}
\Delta\Delta G = \Delta G_s - \Delta G_I &= \Delta G_{E'} - \Delta G_E \\
&= -RTln\frac{K_m/K_i}{K'_m/K'_i}
\end{aligned}
$$

where the last equality results from substituting equations S8 for $\Delta G_{E'}$ and ΔG_E.

Figure S1 | Probability densities for the energy distributions in DHF bound, **(a)** F153S and **(b)** S153F runs.

(a)

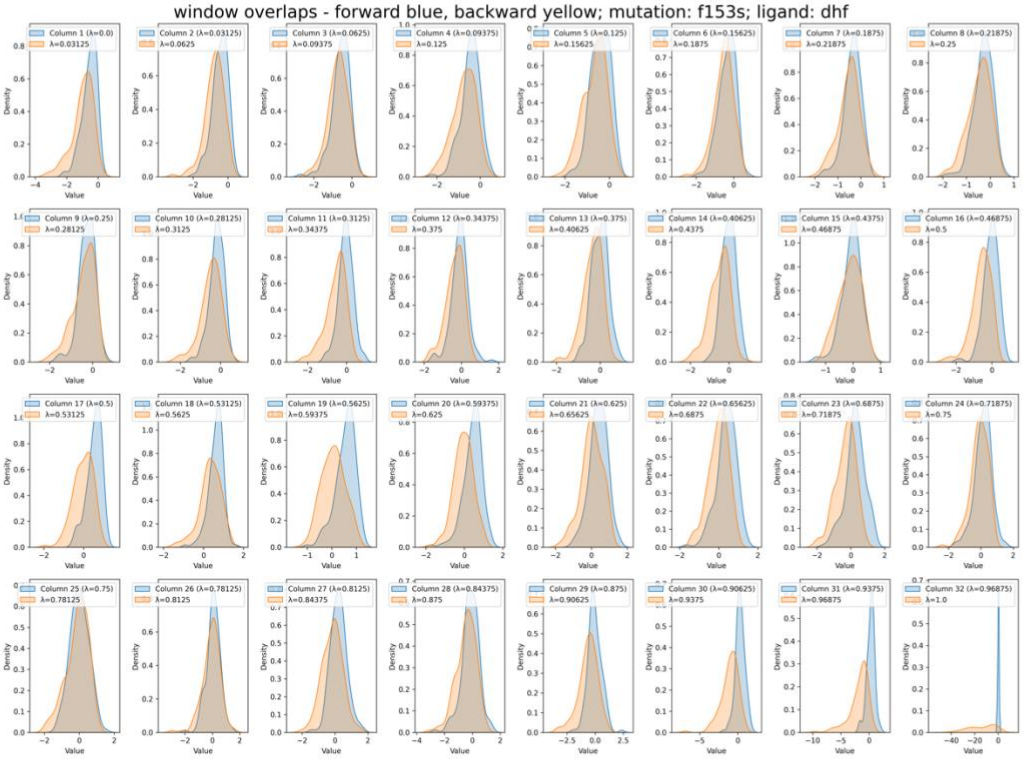

(b)

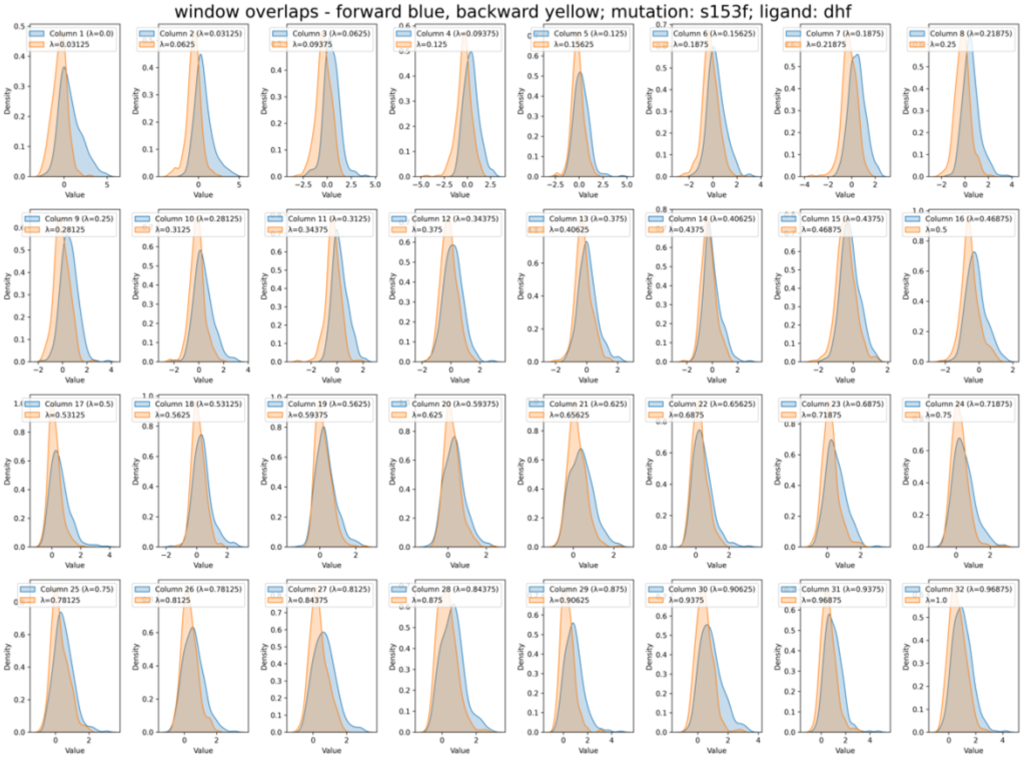

Supplement: Supplementary file 1 — ct5c00193_si_001.pdf [file ct5c00193_si_001.pdf]
